# Investigation of the Genes Involved in the Outbreaks of *Escherichia coli* and *Salmonella* spp. in the United States

**DOI:** 10.3390/antibiotics10101274

**Published:** 2021-10-19

**Authors:** Michelle Li, Kyle Wang, Ashley Tang, Aaron Tang, Andrew Chen, Zuyi Huang

**Affiliations:** Department of Chemical and Biological Engineering, Villanova University, Villanova, PA 19085, USA; michelle46074@gmail.com (M.L.); kylewang122205@gmail.com (K.W.); ashleytangthe1st@gmail.com (A.T.); tangaaron18@gmail.com (A.T.); andew73885@gmail.com (A.C.)

**Keywords:** outbreak, *Escherichia coli*, *Salmonella* spp., NCBI Pathogen Detection Isolates Browser, principal component analysis, hierarchical clustering

## Abstract

*Salmonella* spp. and *Escherichia* *coli* (*E. coli*) are two of the deadliest foodborne pathogens in the US. Genes involved in antimicrobial resistance, virulence, and stress response, enable these pathogens to increase their pathogenicity. This study aims to examine the genes detected in both outbreak and non-outbreak *Salmonella* spp. and *E. coli* by analyzing the data from the National Centre for Biotechnology Information (NCBI) Pathogen Detection Isolates Browser database. A multivariate statistical analysis was conducted on the genes detected in isolates of outbreak *Salmonella* spp., non-outbreak *Salmonella* spp., outbreak *E. coli*, and non-outbreak *E. coli*. The genes from the data were projected onto a two-dimensional space through principal component analysis. Hierarchical clustering was then used to quantify the relationship between the genes in the dataset. Most of the outlier genes identified in *E. coli* isolates are virulence genes, while outlier genes identified in *Salmonella* spp. are mainly involved in stress response. Gene *epeA*, which encodes a high-molecular-weight serine protease autotransporter of Enterobacteriaceae (SPATE) protein, along with *subA* and *subB* that encode cytotoxic activity, may contribute to the pathogenesis of outbreak *E. coli*. The *iro* operon and *ars* operon may play a role in the ecological success of the epidemic clones of *Salmonella* spp. Concurrent relationships between *esp* and *ter* operons in *E. coli* and *pco* and *sil* operons in *Salmonella* spp. are found. Stress-response genes (*asr*, *golT*, *golS*), virulence gene (*sinH*), and antimicrobial resistance genes (*mdsA* and *mdsB*) in *Salmonella* spp. also show a concurrent relationship. All these findings provide helpful information for experiment design to combat outbreaks of *E. coli* and *Salmonella* spp.

## 1. Introduction

Foodborne pathogens pose a dangerous risk to public health, as it is estimated that those pathogens cause 76 million cases of illness, 323,000 hospitalizations, and 5000 deaths annually in the US [1]. *Escherichia coli* (*E. coli*) and *Salmonella* spp. are among the most prevalent outbreak pathogens that cause major health issues (e.g., diarrhea, cramps, vomiting, and severe illnesses like hemorrhagic colitis) [2]. In particular, *Salmonella* spp. are involved in outbreaks that are mainly caused by contamination of a wide range of foods, from tomato [3] to cheese and beef [4]. Enterohemorrhagic *E. coli* (e.g., *E. coli O157*) can contaminate water and soil, which may lead to outbreaks. During the Walkerton *E. coli* outbreak, seven people died and 2000 more contracted the sickness [5]. In a different study, scientists observed a strain of *E. coli* that produces a specific kind of cytotoxin (called verocytotoxin) in an outbreak with a surprisingly high risk of hemolytic uremic syndrome [6].

One potential reason for *Salmonella* spp. and *E. coli O157* to cause outbreaks is that these pathogens are resistant to commonly used antibiotics [7]. In an outbreak where 226 out of 252 isolates of *Salmonella* spp. were detected, Newport infections were reported with certain forms of antimicrobial resistance to trimethoprim-sulfamethoxazole, tetracycline, and chloramphenicol, and decreased susceptibility to azithromycin [3]. It is thus important to quickly sequence pathogens and investigate the genes involved in antimicrobial resistance [7]. Extensive studies have been conducted to detect and combat *Salmonella* spp. and *E. coli O157*. When an outbreak takes place, the pathogen isolates generally go through the following analysis steps: serotyped and phage typed analysis, antimicrobial susceptibility testing, and genomic DNA sequencing. Whole genome sequence (WGS) analysis and microarray analysis are among the most used approaches for gene identification (references [8,9] for *Salmonella* spp.; references [10,11,12,13,14] for *E. coli*). Once the activity levels of genes are quantified, bioinformatics approaches are applied to characterize the pathogens’ genotypes. Belgian National Reference Laboratory of Foodborne Outbreaks (NRL-FBO) developed a bioinformatics platform to find all available antimicrobial resistance genes and identify plasmids in the WGS sequences [8]. During a *Salmonella enterica* serovar Enteritidis outbreak in Massachusetts in 2018, a reference-free bioinformatics pipeline was used to analyze WGS data, allowing the creation of a phylogenetic tree to illustrate the relatedness between isolates [15]. Furthermore, a variety of other data analysis methods have been investigated. A team at National Center for Toxicological Research used ArrayTrack as the platform for data analysis for 69 *Salmonella* spp. [9]. Furthermore, a software package, consisting of five types of bioinformatics approaches (pulsed-field gel electrophoresis (PFGE) band standardization, *Salmonella* spp. serotype prediction, hierarchical cluster analysis, distance matrix analysis, and two-way hierarchical cluster analysis), has been developed and integrated with PFGE database to enhance the data mining of PFGE fingerprints [16].

The advance in sequencing and bioinformatics techniques accelerated the discovery of genes involved in antimicrobial resistance, stress response, and virulence of both *Salmonella* spp. and *E. coli O157*. A report in 2020 found heavy metal resistant genes, disinfectant resistance genes, and antimicrobial resistance genes in *E. coli* and *Salmonella* spp. isolated from chicken broiler farms and retail meat [17]. Another study analyzed antimicrobial resistance patterns and virulence genes for the Avian pathogenic *E. coli* (APEC) from broiler chicken farms in Jordan [18]. A third study in 2014 reported that *emrE*, *sugE(c)*, *mdfA*, and *ydgE*/*ydgF* are the most abundant disinfectant resistance genes in *E. coli* from retail meat in the US [19]. These studies, in addition to a few others, highlight examples of extensive research on the antimicrobial resistance genes identified in *E. coli* and *Salmonella* spp. [20,21,22,23,24,25,26,27,28]. However, since more of these existing studies mainly focused on one pathogen (i.e., *E. coli* or *Salmonella* spp.) isolated from one type of source (e.g., beef and water), it is necessary to conduct a comprehensive study on genomic data of multiple pathogens isolated from various sources over time. Expanding upon previous studies, the aim of the study is to study the genomic data of both *E. coli* and *Salmonella* spp. isolated from various sources during outbreak or non-outbreak times in the US.

Before knowledge about antibiotic resistance was prevalent, humans used antibiotics as a convenient cure to life-threatening bacterial infections. However, as humans continue to overuse antibiotics, resistant bacteria flourish, preserving their genes for future generations until the entire population has a resistance to the antibiotics [7]. In addition to the adaptation to antibiotics, pathogens have genes that can readily adapt, over a few generations, to various environmental stresses, resulting in outbreaks from these bacteria that are difficult to contain. Pathogens have responses that make them resilient to changing circumstances with responses that help them survive in a hostile environment, such as acidity, high salt concentrations, and extreme temperatures [29]. It is thus important to evaluate the involvement of stress response genes in outbreak pathogens. After pathogens survive from antimicrobial treatments and hostile stress environments, virulence factors are required for pathogens to infect hosts and then create a niche there ([30,31]). While extensive studies of the outbreaks of *Salmonella* spp. and *E. coli O157* have been conducted to detect pathogens and identify antimicrobial resistance genes, the relationship between the genes involved in antimicrobial resistance, virulence factors, and stress response have not been thoroughly studied for these pathogens. In addition, the comparison of these three types of genes between outbreak and non-outbreak isolates has not been conducted. To address these problems, this study strives to identify and isolate genes related to antimicrobial resistance, virulence and stress responses that would explain the pathogenicity of *Salmonella* spp. and *E. coli O157*. These genes may become good targets for inhibition to suppress the rates of infection of these harmful foodborne pathogens.

The National Centre for Biotechnology Information Pathogens Isolates Browser (NPIB) database offers sensitive and rapid surveillance with enhanced methods of laboratory identification and subtyping for foodborne pathogens. In addition, it identifies antimicrobial resistance genes, stress response genes, and virulence genes for each isolate sample [32,33]. Research for each type of gene for microorganisms from the NPIB has been conducted to some extent. As for antimicrobial resistance, genes are mainly involved in antimicrobial-degradation, antimicrobial efflux pumps, and modification of antimicrobial binding targets [32]. It was reported by Cui et al., 2021 that virulence factors facilitate pathogens to infect host cells with adhesin-like proteins, increased iron reception and uptake, and toxin synthesis for host inhibition [33]. As for stress response genes, the following operons/genes are reported as important: the *mer* operon (responsible for the regulation of mercury binding and resistance), the *ars* operon (which mediates arsenic resistance), and *asr* (which regulates an acid shock protein that allows for survival in acidic conditions) [34]. However, these studies are not focused on either outbreak pathogens or the relationship between the three types of genes (i.e., antimicrobial resistance genes, stress response genes, and virulence genes). To address this, the genomic data for *E. coli* and *Salmonella* spp., most available from 2010 to 2021, in the NPIB database, is thoroughly studied in this work. Although the data includes tens of thousands of samples, the data can be filtered into four categories: outbreak *E. coli*, outbreak *Salmonella* spp., non-outbreak *E. coli*, and non-outbreak *Salmonella* spp. Since the dataset for each category of pathogens consists of hundreds of genes and thousands of samples, a multivariate statistical approach, i.e., principal component analysis (PCA) [35,36], is used to project the genes in the dataset into a two-dimensional space. The outlier genes, which stand out from the bulk of genes due to their occurrence patterns, are identified as important genes. These genes are then analyzed with the hierarchical clustering approach [37,38,39] to investigate the relationship of antimicrobial resistance genes, stress response genes, and virulence genes. The investigation of various types of genes in *Salmonella* spp. and *E. coli* may reveal how these genes collaborate to enhance the antimicrobial resistance, the probability of surviving long enough to reproduce, and ability to cause damage to a host. The genes identified from this work may be used as targets for creating substances to counter *Salmonella* spp. and *E. coli* outbreaks.

## 2. Results

The outlier genes were first identified via the PCA and hierarchical clustering approach as shown in the Materials and Methods section. Those outlier genes were further analyzed using a hierarchical clustering approach to show their similarity in the detection pattern in outbreak *E. coli*, non-outbreak *E. coli*, outbreak *Salmonella* spp., and non-outbreak *Salmonella* spp. These outlier genes were also categorized into antimicrobial resistance genes, stress response genes, and virulence genes to study the similarity between these three groups of genes in detections. These genes were also compared between outbreak and non-outbreak pathogens to find genes specific to outbreak pathogens.

### 2.1. Analysis and Comparison of Genes Detected in the Outbreak and Non-Outbreak E. coli

**Figure 1:**
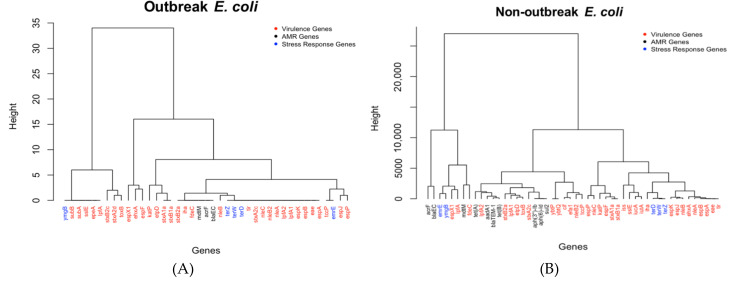
The dendrograms of outlier genes identified for (**A**) outbreak *E. coli* isolates and (**B**) non-outbreak *E. coli* isolates.

The outlier genes for outbreak and non-outbreak *E. coli* are shown in Figure 1A,B in the format of hierarchical dendrograms. The functions of all these important (outlier) genes can be found in Table S1 (Supplementary Material) as a supplemental material. Some notable important genes identified from outbreak *E. coli* (Figure 1A) are *stx* operon genes (i.e., *stxB2c*, *stxA2d*, and *stxB1a*), *esp* operon genes (i.e., *espJ*, *espF*, *espP*, *espX1*, and *espX1*), *nle* operon genes (i.e., *nleA*, *nleB*, *nleC*, and *nleB2*), and a few other genes (e.g., *etpD*, *katP*, and *ehxA*). All these genes are virulence genes that secrete various proteins to efficiently infect host cells and harm the victim. In particular, the *stx* operon genes are involved in the functions for *E. coli* to produce Shiga toxins [40] that can damage small intestines and lead to diarrhea. The *esp* operon in *E. coli* is controlled by a promoter, activated upon contact with eukaryotic cells as a virulence factor operon [41]. The four *nle* genes encode secreted effectors, which are proteins secreted by the bacteria into the host cell that increase pathogenicity [42]. As shown in Figure 1B, most of these virulence-related genes also play an important role in non-outbreak *E. coli*. The important stress response genes remain the same for both outbreak and non-outbreak *E. coli*. Compared to outbreak *E. coli*, there are more antimicrobial resistance genes and stress response genes in the non-outbreak *E. coli*. Genes, such as the *tet* operon genes that encode a tetracycline efflux pump, were commonly detected in non-outbreak *E. coli*.

**Figure 2:**
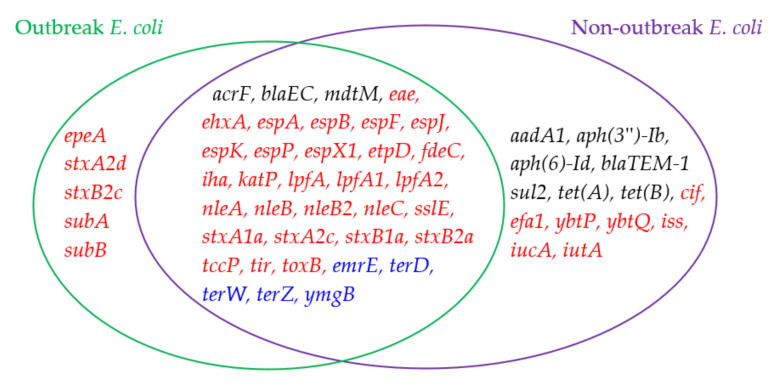
A comparison of outlier genes detected in outbreak *E. coli* and non-outbreak *E. coli*.

While genes from both outbreak *E. coli* and non-outbreak *E. coli* are involved in antimicrobial resistance, stress response, and virulence, the genes in Figure 1A,B are further compared to identify the genes unique to outbreak *E. coli*. Figure 1 shows that genes related to virulence factors outnumber the other two types of genes for both non-outbreak and outbreak *E. coli*. It seems that there is an abundance of virulence genes (marked in red in the figures) in outbreak *E. coli*, which accounts for 33/41 of the total important genes. This ratio is greater when compared to that of non-outbreak *E. coli* (i.e., 35/50). Among these virulence genes, five of them, i.e., *epeA*, *stxA2d*, *stxB2c*, *subA*, and *subB*, are unique to outbreak *E. coli* (Figure 2). The *epeA* gene encodes Enterohemorrhagic *E. coli* (EHEC) plasmid-encoded autotransporter that may impose toxic effect on host cells, such as HeLa cells [43]. While some *stx* operon genes are present in both outbreak and non-outbreak *E. coli*, the outbreak *E. coli* isolates were commonly detected with two extra genes (i.e., *stxA2d* and *stxB2c*) that target the proteins of host cells as extra virulence, making the *E. coli* stronger for an outbreak. The other proteins like *subB* stress response genes are helpful for outbreak *E. coli*, but not as helpful for non-outbreak *E. coli*. *subA* and *subB* genes encode the prototype of the new *AB_5_* toxin family, which are virulence factors that cause massive mortality across the globe (particularly in children from underdeveloped countries) [44]. Contrary to outbreak *E. coli*, more antimicrobial resistant genes were commonly detected in non-outbreak *E. coli*, such as genes: *aadA1*, *aph(3*″)*-Ib*, *aph(6)-Id*, *blaTEM-1*, *sul2*, *tet*(*A*), and *tet*(*B*). Among these genes, *aadA1* encodes aminoglycoside adenylyltransferase, an antimicrobial resistance enzyme in Gram-negative pathogens. Gene *aph(3*″)*-Ib* encodes aminoglycoside phosphotransferase, and *aph*(6)-*Id* encodes for catalyzing the addition of phosphate from ATP. Both of these proteins catalyze the addition of phosphate from ATP. Gene *blaTEM*-1 encodes ampicillin resistance protein, while *sul2* is associated with dihydropteroate synthase type-1 for the resistance to sulfonamide. Both *tet*(*A*) and *tet*(*B*) encode an efflux MFS transporter for tetracycline resistance.

### 2.2. Analysis and Comparison of Genes Detected in the Outbreak and Non-Outbreak Salmonella *spp.*

**Figure 3:**
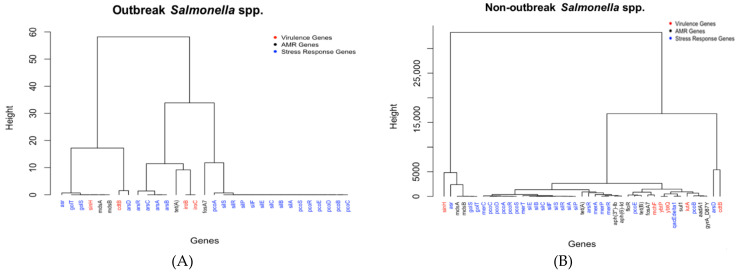
The dendrograms of outlier genes identified for (**A**) outbreak *Salmonella* spp. isolates and (**B**) non-outbreak *Salmonella* spp. isolates.

The important genes identified for outbreak and non-outbreak *Salmonella* spp. are shown in Figure 3A,B, respectively. The important genes detected for outbreak *Salmonella* spp. are mainly from the *iro* operon (e.g., genes *iroB* and *iroC*), the *ars* operon (e.g., genes *arsA*, *arsB*, *arsC*, and *arsR*), the *pco* operon (e.g., genes *pcoA*, *prcoB*, *pcoC*, *pcoD*, *pcoE*, and *pcoR*), and the *sil* operon (e.g., genes *silA*, *silB*, *silC*, *silE*, *silF*, *silP*, *silR*, and *silS*), in addition to a few individual genes such as *tet*(*A*), *fosA7*, and *cdtB*. The *iro* operons are virulence genes (in red in Figure 3A), while the *ars* and *pco* operons are stress response genes that produce a vital enzyme for the survival of *Salmonella* spp. in extreme conditions (in blue in Figure 3B). The three individual genes are two antimicrobial resistance genes, (*tet*(*A*) and *fosA7*), that encode for tetracycline resistance and a virulence gene (*cdtB*) that can help the bacteria create transport proteins that allow them to enter host cells. Contrary to *E. coli*, in which the virulence genes were the major groups in the detected important genes, outbreak *Salmonella* spp. isolates are mainly detected with the stress response genes (24 out of 31 genes). This may be due to that outbreak *Salmonella* spp. undergoes an abundance of stressed conditions, as the bacteria thrives under extreme conditions, such as heat, making it extremely difficult to kill, even if cooked.

Figure 3B represents the relationships between all of the outlier genes detected in non-outbreak *Salmonella* spp. Similar to Figure 3A, certain genes are from operons *pco* and *sil*. The *pco* operon genes, *A*, *B*, *C*, *D*, *E*, *R*, and *S*, all are necessary to code for proteins that are copper resistant, and the *sil* operon genes, *A*, *B*, *C*, *E*, *F*, *P*, *R*, and *S*, are all a part of the *sil* cation-efflux system that causes silver resistance. In addition to operons *pco* and *sil*, certain genes are from the *mer* operon (*mer A*, *C*, *P*, *R*, and *T*) that code for different proteins for mercuric resistance. It is interesting to note that the genes that code for proteins found on the same operon are generally clustered together, with a few exceptions. While most genes were categorized as part of a major cluster, the following four genes stood out when the genes in Figure 3B are clustered into six groups: *cdtB*, *arsD*, *asr*, and *sinH*. The *cdtB* and *sinH* genes are virulence genes (in red) made up of proteins that transport the bacteria into the host cell (i.e., *sinH*) and destroy DNA (i.e., *cdtB*). On the other hand, *arsD* and *asr* are stress response genes coding for proteins that protect the *Salmonella* spp. from extreme conditions.

**Figure 4:**
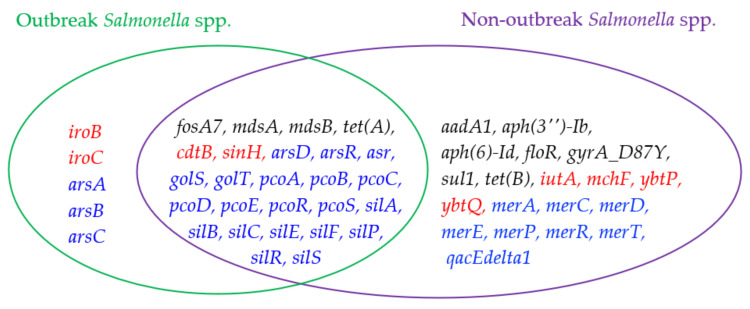
A comparison of outlier genes detected in outbreak *Salmonella* spp. and non-outbreak *Salmonella* spp.

Figure 4 shows a further comparison of the outlier genes commonly detected in outbreak and non-outbreak *Salmonella* spp. (i.e., Figure 3A,B). The following five outlier genes are found mainly in outbreak *Salmonella* spp.: *iroB*, *iroC*, *arsA*, *arsB*, and *arsC*. The *ars* operon genes are stress response genes, which generally include instructions for creating enzymes that make the environment more habitable for *Salmonella* spp. For instance, gene *arsC* converts arsenate (which has been shown to arrest flagellar movement in *Salmonella* spp.) into arsenite [45]. These stress response genes might be helpful for outbreak *Salmonella* spp. to enhance their probability of further survival in extreme conditions. In addition, *iroB* and *iroC* are virulence genes, causing damage to the host cells, making *Salmonella* spp. stronger for causing outbreaks. As for non-outbreak *Salmonella* spp., more antimicrobial resistance genes were uniquely identified as outlier genes. They include *aadA1*, *aph(3*’’)*-Ib*, *aph(6)-Id*, *floR*, *gyrA_D87Y*, *sul1*, and *tet(B)*. It is interesting to find that four of these seven antimicrobial resistance genes were uniquely detected in non-outbreak *E. coli* (when compared to outbreak *E. coli*). While *sul2* was an outlier gene in non-outbreak *E. coli*, *sul1* was the counterpart in non- *Salmonella* spp. *floR* and *gyrA_D87Y* genes are involved in the resistance of florfenicol and fluoroquinolone, respectively. In addition to these antimicrobial resistance genes, the following virulence genes were mainly detected as outlier genes in non-outbreak *E. coli*: *merA*, *merC*, *merD*, *merE*, *merP*, *merR*, *merT*, and *qacEdelta1*. The first seven of these genes are from the *mer* operon for mercuric resistance, and *qacEdelta1* encodes quaternary ammonium compound efflux.

### 2.3. Comparison of Genes Detected in E. coli and Salmonella *spp.*

**Figure 5:**
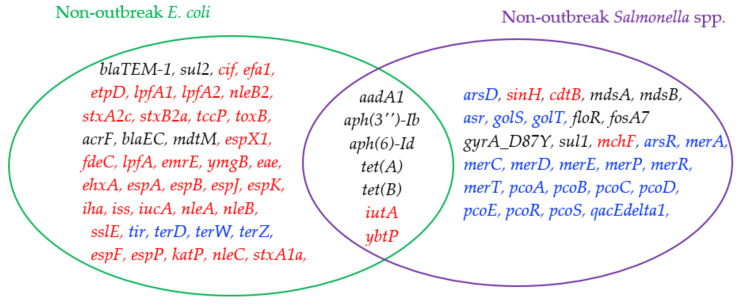
A comparison of outlier genes detected in non-outbreak *E. coli* and non-outbreak *Salmonella* spp.

Since *E. coli* and *Salmonella* spp. are the most common pathogens causing outbreaks, it is of value to compare the outliers detected in them. Interestingly, no outlier gene is shared by outbreak *E. coli* and outbreak *Salmonella* spp. A similar trend is also observed in the comparison of outlier genes between non-outbreak *E. coli* and non-outbreak *Salmonella* spp., which indicates that a relatively low portion of the outlier genes (i.e., seven genes) are shared by them (Figure 5). Five of these genes are antimicrobial genes (i.e., *aadA1*, *aph*(*3*’’)-*Ib*, *aph*(*6*)-*Id*, *tet*(*A*), and *tet*(*B*)), while the other two are virulence genes (i.e., *iutA* and *ybtP*). It is interesting to see that more virulence outlier genes are detected in non-outbreak *E. coli* while more stress-response outlier genes are found in non-outbreak *Salmonella* spp.

## 3. Discussion

### 3.1. Outlier Gene Difference between Outbreak and Non-Outbreak Pathogens

It is important to identify the outlier genes unique to the outbreak pathogens, as these genes may provide useful information related to pathogen outbreaks. As shown in Figure 2, Figure 4 and Figure 5, there are few outlier genes shared between *Salmonella* spp. and *E. coli* in terms of genes. When comparing non-outbreak genes and outbreak genes for the same microorganisms, quite a few similar genes can be identified. Furthermore, 36 genes are shared by outbreak and non-outbreak *E. coli* (Figure 2), while 26 genes are shared by outbreak and non-outbreak *Salmonella* spp. (Figure 4). For the outlier genes shared by the two types of *E. coli*, 28 of them are virulence genes, while three and five of them are antimicrobial resistance genes and stress response genes, respectively. As for *Salmonella* spp., 20 of the 26 outlier genes shared by outbreak and non-outbreak strains are stress-response genes. The rest of the genes are involved in antimicrobial resistance (4 genes) and virulence (2 genes).

While certain outlier genes are shared by outbreak and non-outbreak strains, certain outlier genes are unique to outbreak strains. The following five outlier genes are unique to outbreak *E. coli*: *epeA*, *stxA2d*, *stxB2c*, *subA*, and *subB*. All of them are virulence genes. In particular, gene *epeA* encodes a high-molecular-weight serine protease autotransporter of Enterobacteriaceae (SPATE) protein. It has been reported to contribute to the pathogenesis of Enterohemorrhagic *E. coli* [46]. *stxA2d* and *stxB2c* genes are associated with the *stx* operon that is known for its involvement in the production of one type of *AB_5_* toxins. These toxins may contribute to pathogenesis in certain life-threatening Shiga toxin-producing *E. coli* (STEC) diseases. While other *stx* operon genes are found as outlier genes in non-outbreak *E. coli*, *subA* and and *subB* genes are only regarded as outlier genes for outbreak *E. coli*. Gene *subA* encodes distinct A subunit enzymic activity (i.e., subtilase rather than RNA-*N*-glycosidase or ADP-ribosylase), while a potent cytotoxicity is encoded by *subB* [47]. The cytotoxic activity of these two subtilases may contribute to the pathogenesis of outbreak *E. coli*.

The operon with genes deemed as outliers for outbreak *Salmonella* spp. but not deemed outliers for non-outbreak *Salmonella* spp. is the *iro* operon. The *iro* operon, more specifically *iroB*, is commonly found in *Salmonella enterica Typhi*. Its glycosyltransferase activity is essential for salmochelin production [48]. These virulence genes are critical for *Salmonella* spp. to survive with the necessary level of iron and to increase the pathogenicity for outbreaks. In addition to *iroB* and *iroC*, *arsA*, *arsB*, and *arsC* are the other three genes uniquely deemed as outliers for outbreak *Salmonella* spp. These three genes are associated with the *ars* operon. In addition to them, other *ars* operon genes, such as *arsD* and *arsR*, are also detected as outlier genes for outbreak *Salmonella* spp. These genes encode an essential adaptive feature (i.e., arsenic tolerance) for the ecological success of the epidemic clones of *Salmonella* spp. [49].

### 3.2. Outlier Gene Difference between Salmonella *spp.* and E. coli

Although both *Salmonella* spp. and *E. coli* are Gram-negative bacteria from the Enterobacteriaceae family, there are few outlier genes shared between the two (as shown in Figure 5). Even though *Salmonella* spp. and *E. coli* separated 120 million years ago from a common ancestor, one report found that *Salmonella* spp. and *E. coli* share around 85% of their genomes. Since the divergence of the two bacteria, *Salmonella* spp. have adapted the ability for horizontal gene transfer. The genes encoding *Salmonella* spp. pathogenicity islands are essential for it to survive in extreme conditions [50]. This is true as *E. coli* is usually regarded as a commensal, whereas *Salmonella* spp. is known for causing gastroenteritis and typhoid fever in humans [51]. The variation of the phenotypes is attributed to both point mutations and segments of genomes. Therefore, it is these slight variations in genes that cause distinctive phenotypic characteristics between *Salmonella* spp. and *E. coli* [52]. In particular, the genes in outbreak and non-outbreak *Salmonella* spp. have an abundance of stress response genes. Since *Salmonella* spp. spread to humans if fecal matter is present in food, virulence factors are limited. *Salmonella* spp. do not necessarily infect a host cell by invading it. Instead, *Salmonella* spp. make their way into humans if food is not cooked thoroughly, due to the fact that the stress genes of *Salmonella* spp. enable them withstand extreme conditions, such as abnormal temperature and acidity. Contrary to *Salmonella* spp., outbreak and non-outbreak *E. coli* contain more virulence genes than stress response genes. This may be due to *E. coli*’s commensal role, as this role enables *E. coli* to survive without experiencing as much stress as *Salmonella* spp. On the other hand, the large portion of virulence outlier genes are beneficial for *E. coli* to invade human bodies.

### 3.3. Interaction between Antimicrobial Resistance Genes, Stress Response Genes, and Virulence Genes

Figure 1 and Figure 3 show the dendrograms of outlier genes for *E. coli* and *Salmonella* spp. The three types of genes, i.e., antimicrobial resistance genes, stress response genes, and virulence genes, are listed in different colors. These figures may reveal some hidden interactions between these three types of genes that facilitate the survival of the two pathogens. While it is not surprising to see that genes within the same operon generally stay close to each other in the dendrograms, certain genes from different types/operons stay together. Those genes are discussed separately below for *E. coli* and *Salmonella* spp.

As shown in the dendrograms in Figure 1, the two operons that share the greatest similarities between the results are the *esp* and *ter* operons for both outbreak and non-outbreak *E. coli*. The *esp* operon genes (which include *espX1*, *espA*, *espB*, *espF*, *espJ*, *espK*, and *espP*) are all a part of the type three secretion system. The type three secretion system is the process where pathogenic Gram-negative bacteria transport virulence proteins, known as effectors, straight into the cytosol of the host cells. This function is commonly found in enteropathogenic *E. coli* which belongs in the Shiga toxin-producing *E. coli* (STEC) family [53]. Most STECs also possess tellurite resistance, which is possible through the *ter* operon [54]. With both of these operons found encoded in STECs, similarities between them shown in the data may be due to the prevalence of STECs compared to other pathotypes.

The genes from *pco* and *sil* operons generally stay together in the dendrograms for both outbreak and non-outbreak *Salmonella* spp. (Figure 3). The *sil* operon causes resistance to silver, while the *pco* operon allows for copper resistance. Both the operons are a part of a Tn-7-like structure in *Salmonella* spp. Together they likely have the potential to cause co-selection of antibiotic resistance genes [55]. While *sil* and *pco* operon genes are both stress-response genes, the following six genes, from different gene types, stay in the same dendrogram branch for both outbreak and non-outbreak *Salmonella* spp.: *asr*, *golT*, *golS*, *sinH*, *mdsA*, and *mdsB*. Among them, *asr*, *golT*, and *golS* are stress response genes; *sinH* is a virulence gene; *mdsA* and *mdsB* are antimicrobial resistance genes. In particular, *asr* encodes an acid shock protein that allows for survival in acidic conditions. *GolT* is a P-type ATPase that enables *Salmonella* spp. to detect the presence of gold salts in the environment and to mount the appropriate resistance response. The expression of *GolT* is controlled by *GolS*, an MerR-like sensor that is highly selective for Au ions [56]. These stress-response genes enable *Salmonella* spp. to survive hostile environments. The *sinH* gene encodes an autotransporter protein that facilitates the adhesion and invasion of *Salmonella* spp. into host cells, while *mdsA* and *mdsB* are from the *mds* operon that encodes membrane fusion proteins of the multidrug and metal efflux. In another report, it was interesting to find that the aforementioned six genes are observed in over 90% of endemic and ecdemic non-typhoidal *Salmonella* spp. circulating among animals and animal products in South Africa over a 60-year period [57]. These genes may have hidden interactions that are worthy of further investigation in the future.

## 4. Materials and Methods

### 4.1. Gene Data of E. coli and Salmonella *spp.* from NPIB Database

The gene data from the NPIB database was downloaded as an individual Excel file for each of the four categories: outbreak *E. coli*, outbreak *Salmonella* spp., non-outbreak *E. coli*, and non-outbreak *Salmonella* spp. The following information was contained in each dataset: microorganism (*E. coli* or *Salmonella* spp.), collection year, isolation location (which state in the US), isolation source, isolation types (environmental versus clinical setting), antimicrobial resistance genes, stress response genes, virulence genes, and outbreak information (outbreak or non-outbreak). A MATLAB program has been developed to digitize thousands of samples into a table format (stored in a CSV Excel file) so that the data can be processed in the R programming platform. Each row of the table contains the information for one sample and each column represents one variable. Table 1 shows a portion of the Excel data matrix extracted from the outbreak *Salmonella* spp. dataset. Each row represents one sample and each column represents a gene identified in outbreak *Salmonella* spp. In sample one (i.e., the second row in the table), *Salmonella* spp. was detected in a sample from 2019 (i.e., “organism = 1” for *Salmonella* spp. and “organism = 2” for *E. coli*) in an environmental setting (i.e., “Type = 1” for the clinical setting and “Type = 2” for the environmental setting). The “0” value in the *fosA7* and *tet*(*A*) column indicated that these two gene were not detected in that sample. On the contrary, *mdsA* and *mdsB* genes were detected in the pathogen. Table 1 only lists several of the hundreds of genes for an illustrative purpose. If the pathogen was sampled during an outbreak, a value of “1” was assigned to the column represented by the variable “outbreak”. Otherwise, a “0” value was assigned. Table 1 was imported into the R programming language for data analysis, and the names of the columns in Table 1 were used as the names of variables to recall the values listed in the corresponding columns.

### 4.2. Identification of Important Genes via PCA and Hierarchical Clustering

**Figure 6:**
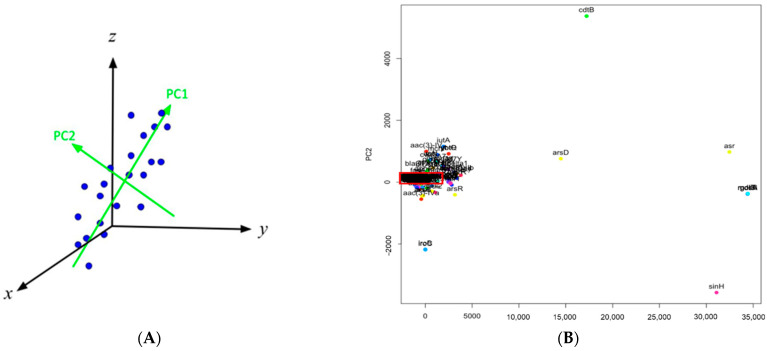
(**A**) illustration on how a three-dimensional space (i.e., *x*-*y*-*z*) is reduced to a two-dimensional space (i.e., PC1–PC2) with principal component analysis, in which the projections of the data points onto the PC1 direction have the largest variance; (**B**) the high-dimensional dataset for non-outbreak *Salmonella* spp. was reduced to the two-dimensional PC1–PC2 space so that the genes are visualized. The red rectangle area is for the bulk genes lumped together.

Principal component analysis is one of the most commonly used unsupervised statistical approaches in which high-dimensional data can be visualized in a two-dimensional space with a good accuracy. To process the data with PCA, a matrix is generated in such a way that each gene corresponds to a row while each column represents the times that gene was detected in samples during each year (from 2010 to 2021). The number of columns in the generated matrix, i.e., 12 columns for 12 years, represent the number of dimensions the matrix contains. It is challenging to project the genes in the dataset in a 12-dimensional space. PCA was thus implemented to reduce the data to a two-dimensional space so that the genes were visualized and further studied via the hierarchical clustering approach for their relationships. In particular, the dimension in PCA that contains the largest amount of variance and provides the strongest rendering of the data is called the first principal component (PC1). Accordingly, the second principal component (PC2) is the direction with the second highest variance and perpendicular to PC1. PC1 and PC2 are generally used to build the data’s reduced-dimensional graph. Figure 6A illustrates the idea on how PCA can be used to reduce data dimension. The PC1 and PC2 directions were identified for the data presented in a three-dimensional space (i.e., *x*-*y*-*z* space). It can be seen that the projections on the PC1 direction show the largest variance. The data points can still be distinguished by their projections onto the PC1–PC2 space. Therefore, the three-dimensional space (i.e., *x*-*y*-*z* space) can be reduced to a two-dimensional space characterized by PC1 and PC2. While Figure 6 provides a general description on how to reduce a three-dimensional space to a two-dimensional space, a similar approach was used in this work to project the genes from a high-dimensional space to a PC1–PC2 space.

The projections of the genes into a PC1–PC2 space can facilitate the identification of clusters that signal similarities between the genes on how they are detected in the pathogens over time. The genes outside the largest cluster are deemed outliers. The clustering helps visualize the patterns within each data set which would otherwise go unseen in a multivariable data set. As an example, Figure 6B shows the genes projected from the dataset for non-outbreak *Salmonella* spp. Since there are 360 genes in that dataset, it is impossible to show the names of all genes clearly in Figure 6B. This figure is mainly used to illustrate the challenge to study the relationship of a large number of genes. In particular, many genes are lumped together in the red rectangle area in Figure 6B. The genes not in that area, especially those outlier genes, show different occurrence patterns from those bulk genes in the red rectangle area. A close examination of detection frequencies of these two groups of genes in non-outbreak *Salmonella* spp., i.e., the bulk genes versus the outlier genes, indicate that the outlier genes are of more apparent occurrence patterns. The outlier genes are then regarded as important for further study on how they are involved in antimicrobial resistance, stress response, and virulence. While the projections on the PC1–PC2 space are helpful for identifying outlier genes, PCA does not return the quantitative relationship of those genes. PCA is not able to show all the genes clearly as the bulk of the genes are lumped together. Therefore, the hierarchical clustering approach is further implemented to illustrate the relationship between those genes and provide a quantitative approach for selecting the outlier genes.

**Figure 7:**
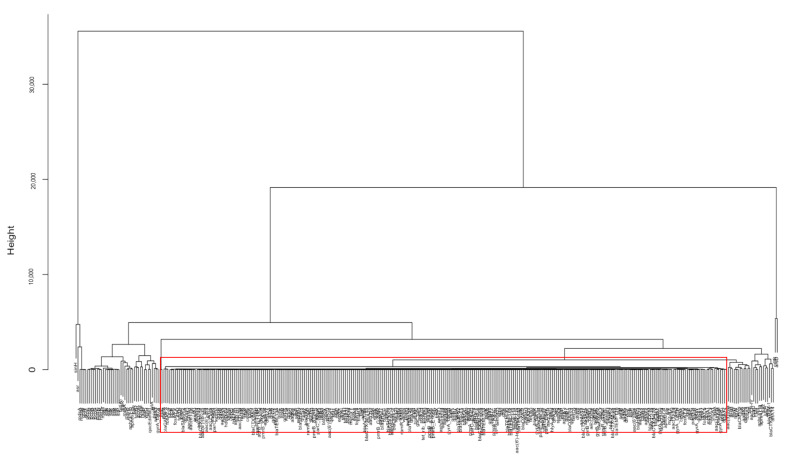
A hierarchical cluster of genes from the dataset for non-outbreak *Salmonella* spp. The genes in the red rectangle correspond to the bulk genes in the red rectangle of Figure 6B. Compared to the results from PCA, the relationship between genes is illustrated in hierarchical clustering.

Hierarchical clustering is used in this work to select similar genes into groups called clusters and illustrate the relationship between genes in the dendrogram format. It differs from K-means clustering, another popular type of unsupervised learning that clusters data points based on similarity, in the sense that in K-means clustering, the number of clusters can be pre-specified so that the genes are specified into *k* clusters, while in hierarchical clustering, the number of clusters is not pre-specified. Hierarchical clustering outputs a dendrogram, a tree-like visual representation of each possible number of clusters, from 1 to *n*, with *n* as the total number of objects in the dataset. Furthermore, hierarchical clustering shows a detailed relationship between the genes, making it more beneficial than K-means clustering. In hierarchical clustering, at the very top of the dendrogram is the most general cluster. Lower on the dendrogram, the genes in the cluster are more similar to one another, compared to those connected on the top. To illustrate this, Figure 7 shows the result of hierarchical clustering for the data from the dataset for non-outbreak *Salmonella* spp. Since there are 360 genes in the dendrogram, the names of individual genes can only be seen by zooming in Figure 7. Similar to Figure 6A, Figure 7 is mainly used to illustrate the general procedure to identify the outlier genes. The identified outlier genes are shown in detail in the Results section. Compared to the PCA result shown in Figure 6B, the relationship between individual genes can be found in the hierarchical dendrogram. The bulk genes lumped together in the red rectangle in Figure 6B can be further identified as the genes in the red rectangle, which contains the largest number of genes showing a similar low occurrence pattern. The other genes (e.g., *asr* and *sinH*) are regarded as outlier genes which are targets for further investigation for their involvement in antimicrobial resistance, stress response, and virulence factors of the pathogen. The outlier genes identified from the hierarchical clustering approach can be confirmed by the PCA result. In Figure 6B, the outlier genes *asr* and *sinH* in Figure 7 stay away from the bulk genes in PCA. As a result, hierarchical clustering is a critical method in this work, as it is able to easily analyze hundreds of genes, observe and visualize which sub-clusters relate to each other, and determine how closely related the genes are using the vertical distance. The outlier genes identified from hierarchical clustering for *E. coli* and *Salmonella* spp. can be further compared to study similarity and dissimilarity in genes during outbreaks and non-outbreaks.

## 5. Conclusions

The historical gene data for the US from National Centre for Biotechnology Information Pathogen Detection Isolates Browser was analyzed with multivariate statistical analysis to identify genes important to outbreak and non-outbreak *E. coli* and outbreak and non-outbreak *Salmonella* spp. The results show that stress-response genes are the major outlier genes for *Salmonella* spp. On the other hand, most outlier genes detected in *E. coli* are virulence genes. This leads to only a few outlier genes shared by *Salmonella* spp. and *E. coli*. Compared to non-outbreak *E. coli*, *epeA*, *stxA2d*, *stxB2c*, *subA*, and *subB* genes were uniquely identified as outlier genes for outbreak *E. coli*. The *iro* operon genes and *ars* operon genes were unique outlier genes to outbreak *Salmonella* spp. Certain antimicrobial resistance genes, stress-response genes, and virulence genes were found to coexist together in the historical data. They include *asr*, *golT*, *golS*, *sinH*, *mdsA*, and *mdsB* genes for *E. coli*, and the *esp* and *ter* operon genes for *Salmonella* spp. The findings from this work may be used to generate hypotheses for further experimental study of outbreak *Salmonella* spp. and *E. coli*.

## Figures and Tables

**Table 1:** A portion of the Excel data matrix for the outbreak *Salmonella* spp. dataset.

Organism	Collection Year	Type	Outbreak	*fosA7*	*mdsA*	*mdsB*	…	*tet*(*A*)
1	2019	2	0	0	1	1	…	0
1	2018	2	0	0	1	1	…	0
1	2016	2	1	0	1	1	…	0
1	2012	2	0	1	1	1	…	0
1	2014	2	0	1	1	1	…	0
1	2012	2	0	1	1	1	…	0

## Data Availability

Data can be provided upon request from the corresponding author.

## Supplementary Materials

The following are available online at https://www.mdpi.com/article/10.3390/antibiotics10101274/s1, Table S1: Functions of outlier genes shown in Figure 1, Figure 2, Figure 3, Figure 4 and Figure 5.
