# Supplementary material for: Investigation of the Genes Involved in the Outbreaks of Escherichia coli and Salmonella spp. in the United States
Source: Antibiotics (Basel). 2021 Oct 19;10(10):1274. doi: 10.3390/antibiotics10101274 (PMC8532668; doi:10.3390/antibiotics10101274)
Supplement: Supplementary file 1 [file antibiotics-10-01274-s001.zip › antibiotics-1396897-supplementary.pdf]

Supplementary Materials

Investigation of the Genes Involved in the Outbreaks of *Escherichia coli* and *Salmonella* spp. in the United States

Table S1. Functions of Outlier Genes Shown in Figures 1–5.

Genes	Function of Genes
aadA1	aminoglycoside adenylyltransferase (resistance enzyme in Gram-negative pathogens)
acrF	multidrug efflux pump
aph(3′′)-Ib	aminoglycoside phosphotransferase (catalyzes the addition of phosphate from ATP)
aph(6)-Id	aminoglycoside O-phosphotransferase (catalyzes the addition of phosphate from ATP)
arsA	provides instructions for making the enzyme arylsulfatase A
arsB	provides instructions for producing an enzyme called arylsulfatase B
arsC	arsenate reductase (reduces arsenate to arsenite)
arsD	arylsulfatase D (protein coding gene)
arsR	family transcriptional regulator (trans-acting regulator protein)
asr	arylsulfatase (regulates an acid shock protein)
blaEC	*beta-lactamase (resistance to antibiotics like penicillin)*
blaTEM-1	*ampicillin resistance protein*
cdtB	cytolethal distending toxin (causes DNA damage in intoxicated cells)
cif	*ubiquitin specific peptidase (gene that process proteins that label other proteins for degradation)*
eae	*intimin (virulence protein that attaches to the host cell)*
efa1	*defensin alpha 1*
ehxA	*calcium-binding region protein*
emrE	*multidrug resistance pump*
epeA	*EHEC plasmid-encoded autotransporter*
espA	*protein espA*
espB	*protein espB*
espF	*espF-like protein*

espJ	*hypothetical protein*
espK	*protein espK-like shape*
espP	*putative exoprotein-precursor (extracellular protein?)*
espX1	*secreted effector that binds to a protein and regulates its activity*
etpD	*secretion protein*
fdeC	*intimin-like adhesin (helps attach protein to hose cell*
floR	Florfenicol (resistance gene for florfenicol)
fosA7	Fosfomycin Resistance Gene
golS	MerR-like sensor that is highly selective for Au ions
golT	P-type ATPase
gyrA_D87Y	fluoroquinolone resistance
iha	*pyrimidine-specific ribonucleoside hydrolase (hydrolyzes certain nucleotides)*
iss	*guanine nucleotide disassociation stimulator*
iucA	*Aerobactin siderophore biosynthesis (forms iron siderophores, compounds, which aid in the process of biosynthesis)*
iutA	*signal recognition particle (receptor)*
iroB	enterobactin C-glucosyltransferase (encodes putative glucosyltransferase)
iroC	gliotoxin/aspirochlorine/mycotoxins biosynthesis cytochrome P450 monooxygenase (multidrug ABC transporter ATP-binding protein)
iutA	encodes the aerobactin siderophore ferric receptor protein
mchF	colicin V synthesis protein (ABC transporter protein)
katP	*potassium channel*
lpfA	*fast skeletal myosin light chain*
lpfA1	*long polar fimbrial protein LpfA v*
lpfA2	*major type 1 subunit fimbrin (pilin)*
mdsA	Mucin-desulfating sulfatase (the membrane fusion protein of the multidrug and metal efflux complex MdsABC)
mdsB	inner membrane transporter of the multidrug and metal efflux complex MdsABC
mdtM	*mdtM*
merA	Mercuric reductase (volatilizing mercury as Hg_0)
merC	Mercuric transport protein MerC (Involved in mercuric ion uptake and binding)

merD	HTH-type transcriptional regulator MerD (DNA binding)
merE	Broad-spectrum mercury transporter MerE (mercury ion transmembrane transporter activity: mediates the transport of both CH3Hg(I) and Hg(II) across the membrane)
merP	Mercuric transport protein periplasmic component (mercury resistance operon protein: specifically scavenges for and binds to mercuric ions)
merR	Mercuric resistance operon regulatory protein (represses transcription of the mer operator)
merT	Mercuric transport protein MerT (transfers mercuric ion from the MerP protein to the MerA protein)
nleA	*unknown protein encoded by cryptic prophage CP-933P*
nleB	*T3SS secreted effector NleB (proteins secreted inserted into the host cell to increase pathogenicity*
nleB2	*T3SS secreted effector NleB*
nleC	*T3SS secreted effector NleC*
pcoA	Copper resistance protein A (Required for copper resistance: oxidase activity)
pcoB	Copper resistance protein B (Required for copper resistance)
pcoC	Copper resistance protein C (Required for copper resistance)
pcoD	Copper resistance protein D (Required for copper resistance)
pcoE	Probable copper-binding protein (Required for copper resistance through the activation of the two-component regulatory system CusS/CusR)
pcoR	Transcriptional regulatory protein pcoR (protein in the two-component regulatory system PcoS/PcoR; [possibly a] transcription factor for the cop operon promoter)
pcoS	Probable sensor protein PcoS (protein in the two-component regulatory system PcoS/PcoR: activates PcoR through phosphorylation
qacEdelta1	Quaternary ammonium compound efflux SMR transporter QacE delta 1: transmembrane transporter activity
silA	Putative cation efflux system protein SilA: part of a three-component cation/proton antiporter in the sil cation-efflux system that causes resistance to silver
silB	Putative membrane fusion protein SilB: part of a three-component cation/proton antiporter in the sil cation-efflux system that causes resistance to silver
silC	Probable outer membrane lipoprotein SilC: part of a three-component cation/proton antiporter in the sil cation-efflux system that causes resistance to silver
silE	Silver-binding protein SilE: component of the sil cation-efflux system that causes resistance to silver
silF	cation efflux system protein silF: component of the sil cation-efflux system that causes resistance to silver

silP	Silver exporting P-type ATPase: component of the sil cation-efflux system that causes resistance to silver
silR	transcriptional regulatory protein SilR: component of the two-component regulatory system SilS/SilR and the sil cation-efflux system that causes resistance to silver
silS	sensor kinase SilS: component of the two-component regulatory system SilS/SilR and the sil cation-efflux system that causes resistance to silver; possible activation of SilR through phosphorylation
sinH	Intimin-like inverse autotransporter protein: cell adhesion and pathogenesis
sslE	*putative secreted and surface-associated lipoprotein mucinase*
stxA1a	Shiga toxin 1a
stxA2c	*shiga toxin 2c*
stxA2d	*shiga toxin 2d*
stxB1a	*encoding subunits A and B of Stx1a*
stxB2a	*focus on the purification of the holotoxin or the separated B-subunit*
stxB2c	shiga toxin 2c subunit B
subA	(RefSeq) histone H2B sub acrosomal variant
subB	(RefSeq) Nt-SubB44, TCIP; cold and drought-regulated protein CORA-like
sul1	Dihydropteroate synthase type-1: resistant to sulfonamide; To produce 7,8-dihydropteroate, it catalyzes the condensation of para-aminobenzoate (pABA) with 6-hydroxymethyl-7,8-dihydropterin diphosphate (DHPt-PP) (H2Pte)
tccP	couples Tir to the actin-cytoskeleton
terD	involved in calcium homeostasis and participates in calcium regulation of a DosR-like regulon in Streptomyces coelicolor.
terW	mediates heavy metal ion resistance to tellurium compounds.
terZ	contribute to the tellurium resistance (Ter) mechanism.
tet(A)	tetracycline efflux MFS transporter: tetracycline resistance protein; MFS transporter
tet(B)	tetracycline efflux MFS transporter: tetracycline resistance protein; MFS transporter
tir	*required for efficient pedestal formation in host epithelial cells during infection.*
toxB	restored full adherence capacity to O157Cu, including production and secretion of the proteins.
ybtP	putative ATP binding protein of ABC transporter: nucleotide binding
ybtQ	ABC transporter ATP-binding protein: nucleotide binding

ymgB	repress biofilm formation in rich medium containing glucose, decrease cellular motility and protect the cell from acid which indicates that YmgB has an important function in acid-resistance.

Black—antimicrobial resistance genes; Blue—stress response genes; Red—virulence genes.
